# Impact of oral melatonin on the electroretinogram cone response

**DOI:** 10.1186/1740-3391-7-14

**Published:** 2009-11-19

**Authors:** Anne-Marie Gagné, Konstantin V Danilenko, Serge G Rosolen, Marc Hébert

**Affiliations:** 1 Centre de Recherche Université Laval Robert-Giffard, Faculty of Medicine, Université Laval, Québec, Canada; 2 Institute of Internal Medicine, Siberian Branch of the Russian Academy of Medical Sciences, Novosibirsk, Russia; 3 Centre de Recherche - Institut de la Vision UMR S968, INSERM-UPMC Paris 6, Paris, France; 4 Clinique Vétérinaire Voltaire, Asnières, France

## Abstract

**Background:**

In the eye, melatonin plays a role in promoting light sensitivity at night and modulating many aspects of circadian retinal physiology. It is also an inhibitor of retinal dopamine, which is a promoter of day vision through the cone system. Consequently, it is possible that oral melatonin (an inhibitor of retinal dopamine) taken to alleviate circadian disorders may affect cone functioning. Our aim was to assess the impact of melatonin on the cone response of the human retina using electroretinography (ERG).

**Methods:**

Twelve healthy participants aged between 18 to 52 years old were submitted to a placebo-controlled, double-blind, crossover, and counterbalanced-order design. The subjects were tested on 2 sessions beginning first with a baseline ERG, followed by the administration of the placebo or melatonin condition and then, 30 min later, a second ERG to test the effect.

**Results:**

Following oral melatonin administration, a significant decrease of about 8% of the cone maximal response was observed (mean 6.9 μV ± SEM 2.0; P = 0.0065) along with a prolonged b-wave implicit time of 0.4 ms ± 0.1, 50 minutes after ingestion.

**Conclusion:**

Oral melatonin appears to reach the eye through the circulation. When it is administered at a time of day when it is not usually present, melatonin appears to reduce input to retinal cones. We believe that the impact of melatonin on retinal function should be taken into consideration when used without supervision in chronic self-medication for sleep or circadian disorder treatment.

## Background

Melatonin is a circulating hormone (N-acetyl-5 methoxytryptamine) produced mostly by the pineal gland at night [1]. Considering that melatonin feeds back to the suprachiasmatic nucleus (SCN), site of the internal clock, where melatonin receptors are also located [2], it is suggested that melatonin may be a regulatory hormone of darkness for the SCN. Of interest, melatonin can also be suppressed by light exposure to the eyes not only in animals but also in humans [3]. Because the production of this hormone is "light sensitive", it is not surprising that it is also produced in the retina by the photoreceptors [4] and that melatonin MT1 receptors have been localized in the mammalian eye including the human eye [5]. In fact, melatonin is produced by many structures of the eye including the lens [6], the iris, the ciliary body [7] and the lacrimal glands [8]. Moreover, there is strong evidence for the existence of an ocular circadian clock in mammals. This implies the possibility of interactions between retinal processes and the SCN, which could represent an important input in the control of circadian rhythms [9]. For example, it was found that the mouse retina possesses its own circadian clock and that it regulates the circadian pattern of melatonin secretion [10] albeit the latter appears to be also under partial control of the pineal gland [11]. Retinal melatonin appears to play a role in promoting light sensitivity at night and modulating many aspects of circadian retinal physiology such as rod disk shedding [12,13]. Beside melatonin, it has been shown in avian retina that dopamine is produced from specific amacrine cells [14] and in a circadian rhythm manner in the rat retina [15]. In various animal models, while melatonin production is known to increase during the night, dopamine production, triggered by light, is produced mostly during the day [16]. In rodents, these two retinal neuromediators appear to act as mutually inhibitory signals [17,18], so that when melatonin level is high dopamine level is low and when dopamine level is high melatonin level is low. Consequently, melatonin is thought to promote night vision (rod pathway)[19] whereas dopamine appears to promote day vision (cone pathway) [16].

Due to the close link between melatonin and the biological clock, studies have been performed to use it as a possible resynchronizing agent to treat jetlag [20] and sleep disorders with doses ranging from as low as 0.03 mg to as high as 85 mg [21]. Toxicity of melatonin administration on retinal health may, however, represent a concern since this hormone seems to impact the susceptibility to light-induced damage of rat's photoreceptors [22,23]. It could also disturb retinal functioning by interfering with normal dopamine levels necessary to enhance day vision. In fact, a study by Emser and colleagues [24] showed that oral administration of 10 mg of melatonin induces a cone response ERG decline in human. Because the latter study used only a single intensity and only a red flash to test cone function, we were interested in investigating the impact of melatonin on the ERG dynamics of the cone response over a wider range of intensities using conventional white light flashes that allows the production of a luminance response function from which retinal sensitivity can be determined.

## Methods

### Sample

The study was performed between January-February 2004 in Novosibirsk, Russia, in twelve participants (6 men and 6 women) aged between 18-52 years (mean ± SEM: 33.4 ± 4.0 y). All participants were in good general health, non smokers, with normal sleep habits and no transmeridian travel during the last two months. The study was approved by the Ethics Committee of the Institute of Internal Medicine SB RAMS. The participants were fully informed of the nature of the study, and informed consent was obtained.

### Design

The study was composed of a placebo-controlled, double-blind, crossover, and counterbalanced-order design. The subjects were tested on 2 sessions separated by 2-7 days (median = 5 days). Each session lasted two hours beginning with a baseline electroretinography (ERG) followed by one of the two treatments (placebo or melatonin) then a second ERG. Half of the subjects began with the placebo whereas half began with 15 mg of melatonin (Natrol^® ^Chatsworth, CA). On the second sessions, the baseline was repeated followed by the other treatment. All sessions were performed between 12:30 and 16:30.

### Recording procedure

Upon their arrival at the laboratory, subjects were dilated with 0.5% Tropicamide to ensure maximal pupil dilation and kept in room light (~100 lux) for at least 30 minutes before the baseline ERG recording. During this time Grass gold disk electrodes filled with Grass EC2 electrode cream were installed on the forehead (ground), external canthi (reference for each eye). Active eye electrodes were DTL fibers (Shieldex 33/9 Thread, Statex, Bremen, Germany) placed deep into the conjunctival bag as previously described [25].

The baseline ERG recording always started between 13:00-15:00 and began with a 20-min light adaptation period during which the subject was exposed to a rod saturating white light background of 32 cd^.^s^.^m^-2^(~105 lux) delivered by a ganzfeld (Color Dome; Espion system, DIAGNOSYS LLC, Littleton, MA) in order to achieve full field stimulation. During that period, 10 bright flashes [intensity: 0.84 log cd^.^s^.^m^-2^] delivered at 1 hertz (Hz) were presented every 5 minutes to monitor light adaptation. After the 20^th ^minutes of the light adaptation period, series of 10-20 flashes (1 Hz frequency) were presented at 7 decreasing intensities ranging from 0.39 log cd^.^s^.^m^-2 ^to -1.45 log cd^.^s^.^m^-2^(Table 1). Immediately after the end of the ERG protocol, subject took five melatonin (total of 15 mg) or placebo pills, and the same ERG protocol was repeated 30 min later. During this 30-min period, the subject was kept in room light (about 100 lux).

**Table 1:** Routine ERG protocol

Period	Time since start, min	Flash intensity, log cd·s·m^-2^	N of flashes	Interval, sec.	Ganzfeld background, cd·s·m^-2^
Adaptation	0	0.84	10	1	32
	
	5	0.84	10	1	32
	
	10	0.84	10	1	32
	
	15	0.84	10	1	32
	
	20*	0.84	10	1	32
	
Testing		0.39	10	1	32
	
		0.10	10	1	32
	
		-0.21	10	1	32
	
		-0.61	10	1	32
	
		-0.90	10	1	32
	
		-1.10	10-20	1	32
	
		-1.45	10-20	1	32

* The response obtained at the 20^th ^minute represents the end of the light adaptation and the beginning of the testing period

The white light flashes (10 μsec in duration) were generated by a tungsten stroboscope driven by a PS22 stimulator (^®^Grass, Quincy, USA). The flash-evoked bio-potential of the retina was recorded with a band pass of 1-1000 Hz, with an amplification of × 10 000 times (BIOPAC amplifiers) and averaged on-line by means of AcqKnowledge 3.7.2 software (RC Electronic Inc., USA).

### Analysis

The typical cone ERG waveform is composed of a negative component called the a-wave followed by a positive component called the b-wave [26]. By convention, the a-wave implicit time is measured from the stimulus onset to the minimum voltage (trough) of the waveform deflection whereas the b-wave implicit time is measured from the stimulus onset to the maximum voltage (peak) of the waveform inflexion. The a-wave amplitude was measured from baseline to trough, and the b-wave from the trough of the a-wave to the peak of the b-wave. Each b-wave amplitude were then plotted against flash intensities in order to generate the luminance response function from which log K parameter was derived [25]. Vmax represents the ERG maximum b-wave amplitude observed on the data points used to generate the luminance response function whereas log K parameter, which is interpreted as retinal sensitivity, represents the flash intensity necessary to achieve half of Vmax amplitude. Finally, oscillatory potentials (OPs; wavelets observed on the ascending branch of the b-wave) were extracted from the maximal ERG response (Vmax) by bandpassing off-line the waveforms between 100-500 Hz. Artifact-free responses (e.g blinks) from the two eyes were averaged prior to analysis.

Analysis of variance for repeated measures (rANOVA) was used to assess the effect of Treatment (melatonin vs. placebo) and Time (before vs. after) on the following ERG parameters: Vmax, a-wave and b-wave amplitude and implicit time, log K and sum of OPs. When the rANOVA yielded a positive result (Huynh-Feldt's corrected P < 0.05), the significant differences were analysed with Student's paired *t*-test. Mean values are accompanied with the standard errors of the means (± SEM).

## Results

**Figure 1:**
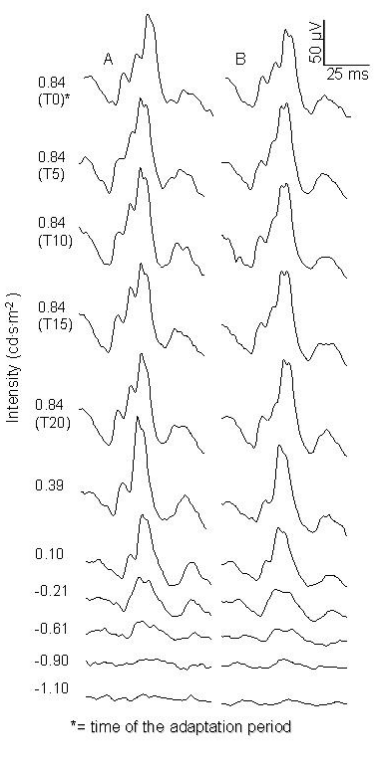
**Raw ERG waveforms obtained from a male (18 y.o) before (A) and after (B) melatonin administration during adaptation and testing period**.

**Figure 2:**
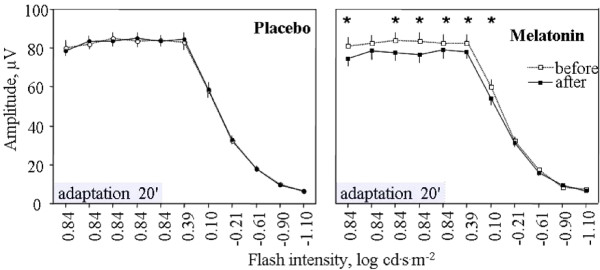
**Changes of the amplitude of ERG response after placebo and melatonin in 12 healthy subjects**. *indicates significant difference (P < 0.05, Student's paired t-test).

Figure 1 shows an example of waveforms obtained before and after melatonin administration during adaptation and testing period at every intensity. In Figure 2, the mean ERG b-wave amplitudes obtained from our protocol are plotted against intensities. It can be seen that data before and after the placebo appear identical, whereas data before and after melatonin appear different for: 1) both the single intensity recorded during the light adaptation period and 2) for the first three highest intensities of the luminance response function. For the adaptation period, rANOVA revealed a significant Treatment by Time interaction for b-wave amplitude (F_1,11 _= 14, P = 0.0032), b-wave implicit time (F_1,11 _= 7.1, P = 0.022) and a-wave implicit time (F_1,11 _= 6.8, P = 0.025). Post-hoc at times 0, 5, 10 and 15 minutes after the beginning of the light adaptation period are presented at Table 2. The b-wave amplitude was significantly decreased after melatonin administration at T0 (7.9 μV ± 1.9 μV), T10 (7.7 μV ± 1.8 μV) and T15 (8.5 μV ± 2.2 μV). This corresponds to a decrease of 10%, 9% and 10% respectively. Also, a significant increase in the b-wave implicit time after melatonin ingestion was found at T10 only (0.4 ms ± 0.2 ms). A-wave implicit time was also significantly longer at T0 and T10. No significant result was found after placebo administration at any adaptation times, for any parameter.

**Table 2:** An immediate change of the ERG indices after melatonin intake

	period	b-wave amplitude	b-wave IT	a-wave IT
	
Adaptation period	T0	P = 0.0014	NS	P = 0.046
	
	T5	NS	NS	NS
	
	T10	P = 0.0014	P = 0.028	P = 0.042
	
	T15	P = 0.003	NS	NS
Testing period	0.84	P = 0.011	P = 0.0061	P = 0.0036
	
	0.39	P = 0.011	NS	NS
	
	0.1	P = 0.0008	NS	NS
	
	-0.21 to -1.1	NS	NS	NS
	
	Vmax	P = 0.0065	P = 0.0003	NS

NS = non significant

For the luminance response function beginning at T20 of the light adaptation period (see Figure 2), rANOVA revealed a significant Treatment by Time Interaction for b-wave amplitude at intensity 0.84 log cd·s·m^-2^(F_1,11 _= 5.2, P = 0.043), 0.39 log cd·s·m^-2^(F_1,11 _= 12.4, P = 0.0048) and 0.1 log cd·s·m^-2 ^(F_1,11 _= 22.4, P = 0.0006). Student post-hoc tests are presented at Table 2, revealed that melatonin administration diminished the amplitude by 6.2% (5.2 μV ± 1.7 μV), 7.5% (6.3 μV ± 2.1 μV) and 9.6% (5.8 μV ± 1.3 μV) at 0.84, 0.39 and 0.1 log cd·s·m^-2^, respectively. Moreover, a significant Treatment by Time interaction was found for the b-wave implicit time (F_1,11 _= 13.9, P = 0.0033) at 0.84 log cd·s·m^-2 ^for which melatonin caused an increased of 0.4 ms ± 0.1 (P = 0.0061). The only significant result for a-wave was a Treatment by Time interaction for the a-wave implicit time at 0.84 log cd·s·m^-2 ^for which melatonin generated an increase of 0.1 ms ± 0.3. No significant result was found after placebo administration at any flash intensity or any parameter.

Because the highest b-wave amplitude did not occur in all subjects at the highest flash intensity (it was observed at 0.39 log cd·s·m^-2 ^in 5 subjects) a separate analysis was performed for the maximal ERG response (Vmax). Significant results were found on the Vmax b-wave amplitude and OP's but not on a-wave or log K parameters. rANOVA revealed a significant Treatment by Time interaction for b-wave amplitude (F_1,11 _= 12.9, P = 0.0043) and implicit time (F_1,11 _= 20.1, P = 0.0009). Melatonin administration caused a significant (see Table 2) decrease of the Vmax amplitude) of 7.9% (6.9 μV ± 2.0 μV) along with an increase of 0.4 ms ± 0.1 ms of the implicit time. The amplitude and implicit time of the three major waves were analyzed: OP2, OP3, and OP4. In some cases (5 participants), only two OPs were detected (OP2 and OP4). However, all the subjects maintained the same number of OPs throughout the study between conditions. Because not all participants demonstrated 3 OPs, we opted to use the sum of OPs for analysis. A significant interaction for Treatment by Time was observed for the Vmax sum of OPs amplitude (F1,11 = 5.51, p = 0.039). This amplitude was reduced after melatonin administration by 6.97 μV ± 2.59 μV (P = 0.023), that is a decrease of 9.9%. The ratio between the Vmax sum of OPs and the respective b-wave amplitude was similar between conditions (F_1,11 _= 0.40, P > 0.5). At Vmax, no significant result was found after placebo administration.

## Discussion

To our knowledge, this is the first study to assess the impact of exogenous melatonin on the human photopic luminance response function. We observed that oral melatonin administration induces a significant decrease of the maximal cone response as well as a decrease of the sum of OPs. The impact of melatonin on retinal function was quite rapid, as it could readily be observed after 30 minutes and persisted for 50 min post ingestion. The decrease in b-wave and sum of OPs were similar in magnitude, with 6.2% to 10% for the b-wave and 9.9% for the sum of OPs. There was no change in cone sensitivity (K index).

The fact that we observed retinal changes in the present study suggests that oral melatonin administration can reach the eye through the general circulation. This is consistent with a previous study using the ERG technique showing that muscular injection of melatonin could have even more impact on fowl's retinal response (reaching the eye through the circulation) than intraocular injection [27].

Because melatonin was administered at a time of day when it is not usually present, we were able to measure its direct impact on cone functioning without any influence of the normal circadian secretion of melatonin in the eye, which occurs at night. Our results are consistent with those of previous studies. For instance, a study performed on the green iguana demonstrated that administration of melatonin during the subjective day reduces the cone b-wave amplitude in a dose-response manner [28]. In humans, an experiment performed by Rufiange et al. [29] showed a strong correlation between the level of salivary melatonin and the ERG cone maximal response, with circadian change in melatonin levels yielding a cone ERG response reduction in the range of 3% to 16%. Also, Emser et al. [19] reported similar results with a decrease of both the scotopic and the photopic human b-wave using 10 mg of exogenous melatonin. However, in the latter study, the scotopic ERG response was triggered by a bright red LED flash, which likely triggered a mixed cone-rod response. In fact, close inspection of the waveforms presented in the paper by the authors show that photopic and scotopic responses were similar both in shape and amplitude. There is therefore a strong suspicion that the findings on the scotopic ERG thus obtained could be attributable to the effect of melatonin on cone functioning.

The way melatonin impacts the cone response is unclear. It has been suggested from the avian model that the presence of melatonin in the circulation could be interpreted by the system as a "night signal" shifting the retina to night vision [11]. But as mentioned earlier, a direct effect on dopamine (especially in day time) must also be considered. Interestingly, a correlation has been found between the dopamine metabolite homovanillic acid (HVA) and the blue cone b-wave amplitude in humans [30]. Among those cocaine-dependant patients, the lower was the presence of HVA in cerebrospinal fluid, the lower was the blue cone b-wave. This is consistent with another study showing that dopamine blockers such as chlorpromazine and fluphenazine can reduce significantly the human ERG cone-dominated response [31].

A mechanism implicating horizontal cells and dopamine could also explain the cone ERG maximal response decrease. These interconnecting neurons help to integrate and regulate the input from multiple photoreceptors. A dopamine reduction due to the presence of melatonin could increase horizontal cell coupling since it has been shown that dopamine antagonists enhance this phenomenon in mudpuppy retina [32]. Knowing that horizontal cells have an inhibitory effect on cones [33], it seems conceivable that increasing horizontal cell coupling (through dopamine inhibition) could provoke a decrease of cone maximal response to light.

However, we cannot exclude the possibility of an alteration of intracellular calcium level by melatonin since this hormone seems to increase the intracellular dispersion of this ion and decrease the gap junction communication between cells. But since this phenomenon has been observed in astrocytes of chick diencephalons, we cannot conclude that melatonin could have the same effect on retinal gap junctions [34].

Regarding the sum of OPs reduction, the origin of this effect is difficult to explain as the exact origin of all OPs is not elucidated yet [35]. In the present study, however, the ratio between the sum of OPs and b-wave amplitude remains similar before and after melatonin, suggesting that OPs were not more affected than the b-wave itself. From our study, we can only conclude that the b-wave and OPs generators are equally affected by melatonin.

Whereas the influence of melatonin on the b-wave was obvious in our study, the effect on the a-wave was not consistent. Since data obtained on primate have shown that the a-wave is generated mostly by photoreceptors [36], this suggests that exogenous melatonin does not have a profound effect on photoreceptors even though MT1 receptors have been found on human cone photoreceptors [37]. However, there is also the possibility of a type II error considering small amplitude-to-noise ratio of a-wave and our small subjects sample.

Although it would have been interesting to perform the same experiment including rods ERG it is unlikely that we would have observed any difference on rods response. In fact, a preliminary melatonin study (oral administration) performed by our group with beagle dogs did not show any significant change on rods ERG with 80 mg of melatonin whereas a substantial effect on cone response was observed [38]. Moreover, we were interested in testing the impact of melatonin during the daytime (when it is sometimes ingested to treat circadian disorders) that is when day vision generated by cones is most significant.

Because melatonin appears to have a negative impact on cone function, we can suspect that naturally increasing melatonin in the evening may play a role in decreasing the impact of the cone system (day vision) in order to better promote the rod system (night vision) and by doing so, ensuring that the most suitable visual function is enhanced according to the time of the day.

## Conclusion

In conclusion, the negative impact of melatonin on cone response may serve to promote night vision. The impact of melatonin on retinal function must be taken into consideration when this compound is used without any supervision in chronic self-medication. Further assessment should be conducted in animal species in order to evaluate a potential effect of chronic use of this compound that is considered as a nutritional (diet) supplement in the United States of America.

## Competing interests

The authors declare that they have no competing interests.

## Authors' contributions

AMG did most of the redaction of the paper and interpretation of the data and contributed to the statistical analysis.

KVD collected the data, contributed to the design of the study and to the statistical analysis.

SGR contributed to the interpretation of the results and the final revision of the manuscript.

MH contributed to the design of the study, the interpretation of the results and performed the final revisions.
